# Prevalence of anxiety and depression among patients with glaucoma

**DOI:** 10.3389/fpsyg.2024.1410890

**Published:** 2024-08-22

**Authors:** Heloisa Helena Abil Russ Giacometti, Laura Fernandes Coelho, Liana Grupenmacher Iankilevich, Livia Stocco Sanches Valentin, Luciana Amizo Ferreira, Marcos Balbino, Regina Cele Silveira Seixas

**Affiliations:** ^1^HR Oftalmologia e Oftalmo Curitiba, Curitiba, Brazil; ^2^Faculdade de Medicina da Universidade de São Paulo, São Paulo, Brazil; ^3^Oftalmo Curitiba, Curitiba, Brazil; ^4^Faculdade de Medicina da Universidade de São Paulo e Instituto do Coração (INCOR), São Paulo, Brazil; ^5^Visclin Oftalmologia, São Paulo, Brazil; ^6^Centro Universitário São Camilo, São Paulo, Brazil; ^7^HCLOE, OPTY, São Paulo, Brazil

**Keywords:** glaucoma, anxiety, depression, GAD-7, PHQ-9

## Abstract

**Purpose:**

This study aimed to determine the prevalence of anxiety and depression among patients with glaucoma compared to the average Brazilian prevalence (9.8% of anxiety and 5.8% of depression, according to the World Health Organization) and its correlation with the severity of the disease.

**Methods:**

This was a transversal, single-arm trial of patients from four glaucoma centers in São Paulo and Curitiba—Brazil. Patients comprised adults at least 18 years of age with glaucoma diagnosis under treatment for at least 6 months. All subjects of the study answered two questionnaires (PHQ-9 and GAD-7) to evaluate the presence of anxiety and depression, and the results were analyzed accordingly to clinical and demographic characteristics.

**Results:**

The protocol included a total of 210 patients. The average age was 61.6 ± 15.3 years, and the female gender was more common (68.86%). Primary open-angle glaucoma was the most common diagnosis (59.90%). The average IOP was 18.5 mmHg, and 1.5 anti-glaucoma drops were the mean treatment. The prevalence of depression and anxiety was 26.90 and 25.71%, respectively. Most patients with anxiety were classified as early glaucoma, while those with depression had severe glaucoma.

**Conclusion:**

This study found that the prevalence of anxiety and depression among patients with glaucoma is higher than in the general population in our country.

## Introduction

Blindness and low vision are the third cause of disability worldwide, according to the Global Burden of Disease (2018). Numerous etiologies can reduce vision, including glaucoma, which causes silent and progressive visual loss and is currently the leading cause of irreversible vision loss worldwide and the second cause of bilateral blindness (Rand Allingham, 2014). It is assumed that 1–2% of population over 40 years old in the world has this disease (approximate 3 millions of people), and in Brazil it is estimated that 1.7 million people are affected, according to the World Health Organization [Associação Paulista de Medicina (APM), 2024].

Glaucoma is a chronic disease affecting the patient’s quality of life and can lead to disabilities in many aspects of life. Its impacts are not only visual loss but also include impairment in daily activities and independent living. It negatively affects social and economic aspects, sometimes associated with depression and anxiety (Wu et al., 2022).

The actual repercussion of glaucoma in stress, anxiety, and depression is yet not entirely determined. Some studies found a negative correlation between glaucoma, anxiety, and depression (Mabuchi et al., 2008, 2012; Rezapour et al., 2018). On the other hand, other studies found a positive correlation between these psychiatric disorders and glaucoma and its severity (Yochim et al., 2012). Mabuchi et al. have estimated that the prevalence of depressive symptoms in glaucoma patients is 10–12% (Mabuchi et al., 2008; Yochim et al., 2012) and that depression was 32% higher in glaucoma patients than in the control group (Skalicky and Goldberg, 2008).

One of the challenges encountered by these studies was to measure in a more objective way how much the Glaucoma impacts the patient’s quality of life. Thus, we chose to use very well studied and validated scales to perform this study, the PHQ-9 (Santos et al., 2013) and GAD-7 (Bártolo et al., 2017), in order to achieve the most reliable result possible.

The Patient Health Questionnaire (PHQ-9) is a test with good validity used for patients and the general population. It has nine questions related to the chronic health illness depression scale. It is validated in Portuguese and is a powerful tool for suspecting depression and assessing response to treatment at the level of depression (Lamela et al., 2020). Generalized Anxiety Disorder (GAD-7) is a quick and reliable tool for anxiety associated with somatic disorders. Also validated in Portuguese, it can be administered by non-clinical personnel; however, a trained clinician must interpret the responses. These questionnaires can also be used over the phone to assess patients with chronic illnesses. AlSwailem et al. (2022), in this study, assessed the level of awareness among ophthalmologists about the need to assess anxiety and depression among patients with chronic eye diseases and the usefulness of the PHQ-9 and GAD-7 for mental health screening.

The primary objective of this study was to analyze the prevalence of depressive and/or anxiety disorders in a group of Brazilian patients, from four reference centers in the southeast of Brazil, diagnosed with glaucoma and to identify its correlation with the severity of the disease, using the PHQ-9 and GAD-7 instruments.

## Methods

### Study design

This is a transversal, single-arm trial, specifically targeted glaucoma patients. It rigorously analyzed the disease’s progression, meticulously gathered data from each patient, compared this data with findings in the scientific literature, and assessed the level of anxiety and depression in the volunteers, establishing clear correlations between these symptoms and the severity of glaucoma. Two hundred and ten patients met the inclusion criteria and were invited to participate. The study involved the glaucoma clinics of two treatment centers in São Paulo (HCLOE and Visclin Oftalmologia) and two in Curitiba (HR Oftalmologia and Oftalmo Curitiba Hospital da Visão/Oftalmoclínica Curitiba). The authors compared the prevalence of depression and anxiety in patients with glaucoma with the prevalence described in the literature.

### Inclusion criteria

The protocol included patients aged 18 years or older without cognitive disorders that could interfere with data collection through the questionnaire and who have had glaucoma with at least one drug treatment for at least 6 months.

### Exclusion criteria

The researchers excluded patients under 18 years of age or with cognitive impairment that may interfere with data collection using standardized questionnaires and patients diagnosed with glaucoma for less than 6 months or without a therapeutic regimen for the disease. Patients with previous diagnosis of anxiety or depression, or diagnosed with any other disease were not excluded, what could potentially interfere with the results.

### Sample size calculation

Few studies describe that the prevalence of depression among patients with glaucoma ranges between 6.6% (The Gutenberg Health Study) (Rezapour et al., 2018), 12.2, 30% (Singaporean study) (Lim et al., 2016), and 41.8% (Southeast Nigeria study) (Onwubiko et al., 2020). According to the World Health Organization (2017), 5.8% of the Brazilian population has depression, and 9.3% has anxiety.

The authors assumed a general prevalence of depression of 5.8% of the Brazilian population (World Health Organization, 2017). Using a two-tailed z-test of proportions with an alpha error of 0.05% and power of 90% to compare this observed Brazilian prevalence with a theoretical prevalence of 30% in patients with glaucoma, we would need 51 patients to detect such a difference between both populations. We intended to be at least 10% above this estimate to compensate for losses to follow-up during the study, so we expected to gather a group of at least 56 patients with glaucoma. We chose the Singapore study due to its intermediate prevalence of depression among the other studies (30%).

### Evaluation of anxiety and depression

All patients were asked to sign the informed consent, and those who agreed to participate responded to two predetermined instruments that assess the presence of anxiety and depression in the individual. The instruments chosen by the researchers are the Patient Health Questionnaire (PHQ-9) (Appendix 1A) and the General Anxiety Disorder (GAD-7) (Appendix 2A), both culturally validated for the Brazilian Portuguese language (Appendices 1B, 2B). They were scoring grids used with the chosen scales (Appendix 3).

The questionnaires were applied to glaucoma patients by researchers, after approval by the ethics and research committee. Each institution participated in remote training from the coordinating center to ensure uniformity in sample collection.

During the analysis of the results, the patients were divided into subgroups according to the severity of the underlying disease (glaucoma) in early, moderate or severe glaucoma (according to clinical criteria for evaluating the visual field that considers the evaluation by Mean Deviation—MD—Hodapp et al., 1993).

Additionally, we analyzed the correlation of the presence of anxiety or depression with the severity of glaucoma and their correlation with therapy (number of eye drops/anti-glaucomatous drugs and invasive procedures such as anti-glaucomatous surgeries).

The following variables were documented: disease severity, sex, age, socioeconomic factors, profession, education, smoking status, systemic diseases, previous surgeries, and the number of eye drops.

For the staging of the disease, we will consider glaucoma as early, moderate and severe, according to the Hodapp-Parrish-Anderson criteria. The visual field features used to diagnose glaucoma are based on the Hodapp-Parrish-Anderson criteria. These criteria include a glaucoma hemifield test that falls outside the normal limits in at least two fields, as well as three or more clusters of non-edge points in a location typical for glaucoma. It is based on the Mean Deviation (MD) data of the perimetry test performed on a Humprey HFA-750 i Zeiss exam. By convention, it is known that early glaucoma has MD ≤ 6 dB, moderate glaucoma MD 6–12 dB and severe glaucoma has MD ≥ 12 dB (Hodapp et al., 1993).

### Statistics

The study aimed to compare the proportion of depression in the general population according to literature data with the proportion found in patients with glaucoma in four ophthalmology centers in Brazil. The protocol stratified the patients according to glaucoma severity and compared subgroups to each other. The distribution of groups was evaluated using Shapiro–Wilk normality tests. We compared means with the t-test for independent samples, if the samples had normal distribution, or the Wilcoxon test if the samples did not pass the normality distribution test. To compare categorical variables and association relationships, we used relative risk and odds ratio to quantify effects and the Chi-square test to compare independent samples. The authors used the STATA 13 program (StataCorp. 2013. Stata Statistical Software: Release 13. College Station, TX: StataCorp LP.) to evaluate the statistic.

## Results

Two hundred and ten volunteers were included in this study. The average age was 61, and females were the most prevalent gender (Table 1). Table 2 shows that most volunteers had a university degree (42.86%), followed by those who only completed elementary school (30.05%). Patients with systemic arterial hypertension accounted for 40.38% of the patients in the sample, and 25.85% had Diabetes Mellitus.

**Table 1:** Demographic data regarding glaucoma staging and gender.

	Glaucoma type			Total	*p*-value
	Early	Moderate	Severe		
Number of eyes, *n* (%)	89 (42.4)	41 (19.5)	80 (38.1)	210 (100.0)	
Age (mean ± SD)	60.06 ± 13.17	64.76 ± 13.97	61.77 ± 17.80	61.63 ± 15.25	0.26
**Gender, *n* (%)**					
Female	61 (68.54)	21 (51.22)	50 (62.50)	132 (68.86)	0.16
Male	28 (31.46)	20 (48.78)	30 (37.50)	78 (37.14)	
**PHQ-9, *n* (%)**					
≤ 10 (no depression)	65 (73.03)	34 (82.93)	56 (70.00)	155 (73.81)	0.30
> 10 (depression)	24 (26.97)	7 (17.07)	24 (30.00)	55 (26.19)	
**GAD-7, *n* (%)**					
< 10 (no anxiety)	59 (66.29)	34 (82.93)	63 (78.75)	156 (74.29)	0.07
≥10 (anxiety)	30 (33.71)	7 (17.07)	17 (21.25)	54 (25.71)	
**Total, *n* (%)**	89 (100.00)	41 (100.00)	80 (100.00)	210 (100.00)	

Mean and standard deviation with appropriate statistical significance level considering *p* < 0.05.

**Table 2:** Systemic diseases and educational level correlated with glaucoma staging.

	Glaucoma type			Total	*p*-value
	Early	Moderate	Severe		
Number of eyes, *n* (%)	89 (42.4)	41 (19.5)	80 (38.1)	210 (100.0)	
**Schooling, *n* (%)**					
Illiterate	2 (0.99)	0 (0.00)	2 (0.99)	4 (1.97)	0.24
Elementary	27 (13.30)	6 (2.96)	28 (13.79)	61 (30.05)	
Middle school	20 (9.85)	12 (5.91)	15 (7.39)	47 (23.15)	
College	37 (18.23)	20 (9.85)	30 (14.78)	87 (42.86)	
Master	0 (0.00)	1 (0.49)	0 (0.00)	1 (0.49)	
Doctorate	1 (0.49)	0 (0.00)	2 (0.99)	3 (1.48)	
**Hypertension**					
No	54 (25.96)	24 (11.54)	46 (22.12)	124 (59.62)	0.95
Yes	35 (16.83)	16 (7,69)	33 (15.87)	84 (40.38)	
**Diabetes**					
No	68 (33.17)	25 (12.20)	59 (28.78)	152 (74.15)	0.17
Yes	21 (10.24)	15 (7.32)	17 (8.29)	53 (25.85)	

The diagnosis of Primary Open Angle Glaucoma was the most common (59.9%); the average anti-glaucoma medication use was 1.68 ± 1.25 medication, and the mean IOP was 18.73 ± 8.09 mmHg.

Regarding the severity stratification, 42.4% of the patients were classified as early, 19.5% as moderate, and 38.1% as severe glaucoma.

The prevalence of depression was 26.19%, and the prevalence of anxiety was 25.71%. In Table 3, we note the correlation between the type of glaucoma identified in our study sample and the disease’s staging.

**Table 3:** Correlation between glaucoma subtypes and glaucoma severity.

	Glaucoma type			Total	*p*-value
	Early	Moderate	Severe		
**PHQ-9, *n* (%)**					
≤ 10 (no depression)	65 (41.94)	34 (21.94)	56 (36.13)	155 (100.00)	0.30
> 10 (depression)	24 (43.64)	7 (12.73)	24 (43.64)	55 (100.00)	
**GAD-7, *n* (%)**					
< 10 (no anxiety)	59 (37.82)	34 (21.79)	63 (40.38)	156 (100.00)	0.07
≥10 (anxiety)	30 (55.56)	7 (12.96)	17 (31.48)	54 (100.00)	

Among patients with Depression by PHQ-9 (scores ≥10), the mean age was 59.81 ± 17.14, the number of anti-glaucoma medications in use was 1.76 ± 1.36 drops, and the mean intraocular pressure was 19.45 ± 8.15 mmHg (Table 4).

**Table 4:** Initial eyedrops and initial intraocular pressure correlated with glaucoma staging.

	Glaucoma type			Total	*p*-value
	Early	Moderate	Severe		
Number of eyes, *n* (%)	89 (42.4)	41 (19.5)	80 (38.1)	210 (100.0)	
Initial eyedrops (number of drugs, mean ± SD)	1.22 ± 1.00	1.68 ± 1.06	2.19 ± 1.40	1.68 ± 1.25	0.001
Initial IOP (mmHg, mean ± SD)	18.11 ± 5.31	116.54 ± 5.79	20.61 ± 10.98	18.73 ± 8.09	0.02

**Figure 1:**
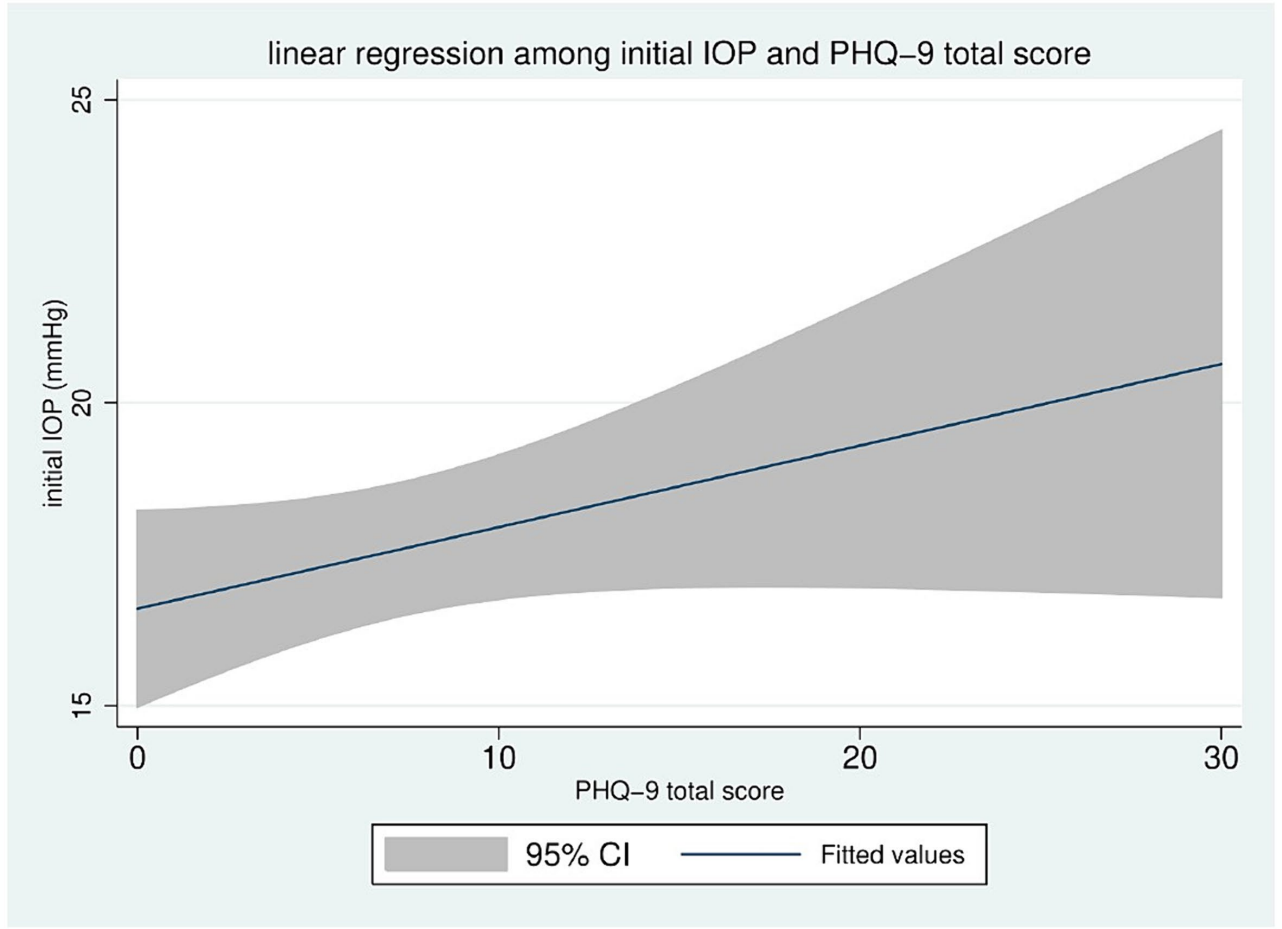
Linear regression among IOP and PHQ total score.

The linear regression between the initial IOP and the total PHQ-9 score shows a positive correlation; as the PHQ-9 score increases, the initial IOP correspondingly increases (Figure 1).

**Figure 2:**
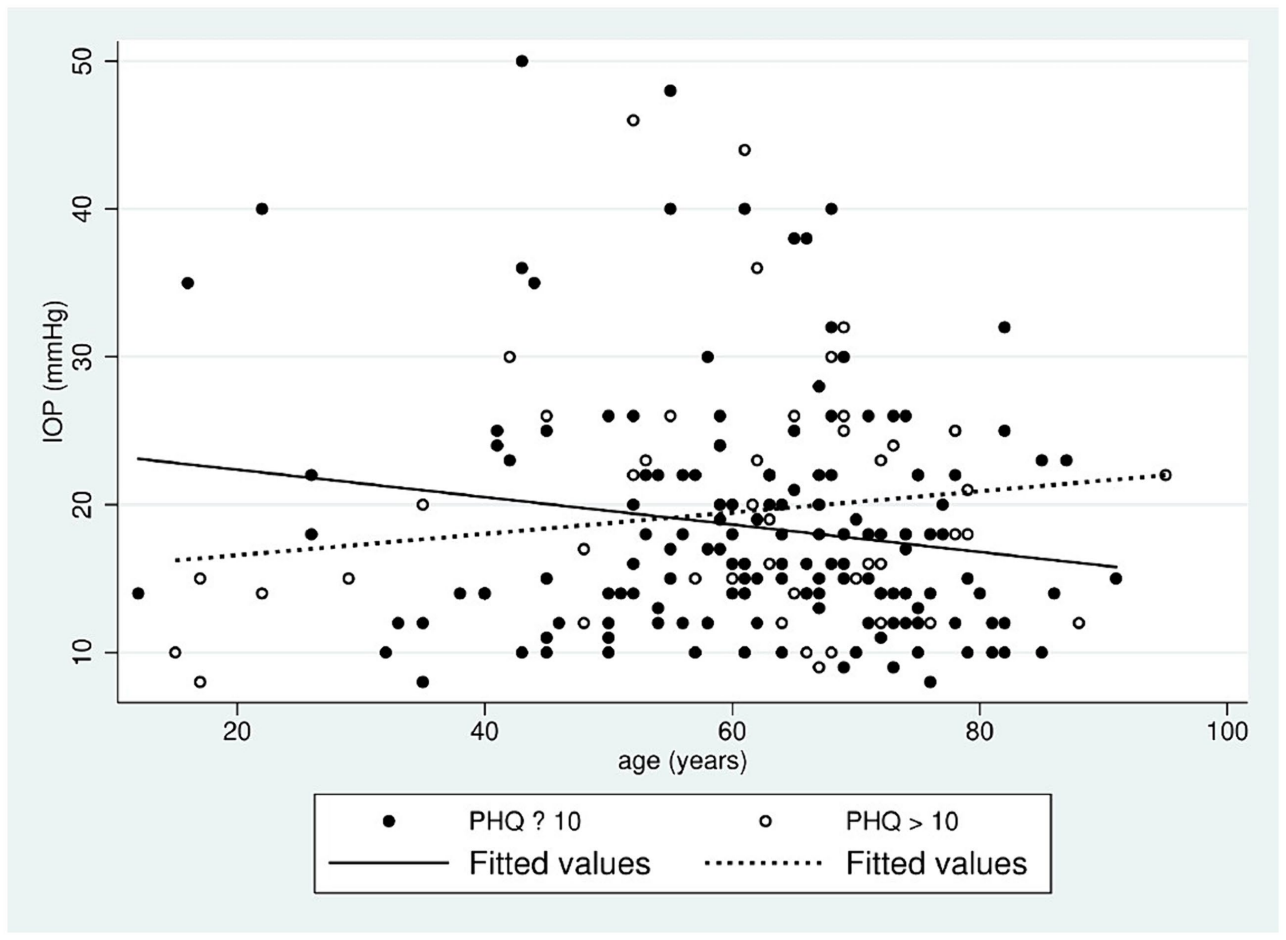
Linear correlation between age and IOP with PHQ in two different levels.

The authors plotted the linear correlation of IOP and age in two groups concerning PHQ-9 scores (≤ 10 and > 10). In patients with PHQ ≤ 10, the correlation is negative, and IOP tends to decrease with age; but in patients with a tendency to depression (PHQ > 10), the correlation is positive, with a tendency for IOP to increase with age (Figure 2).

**Figure 3:**
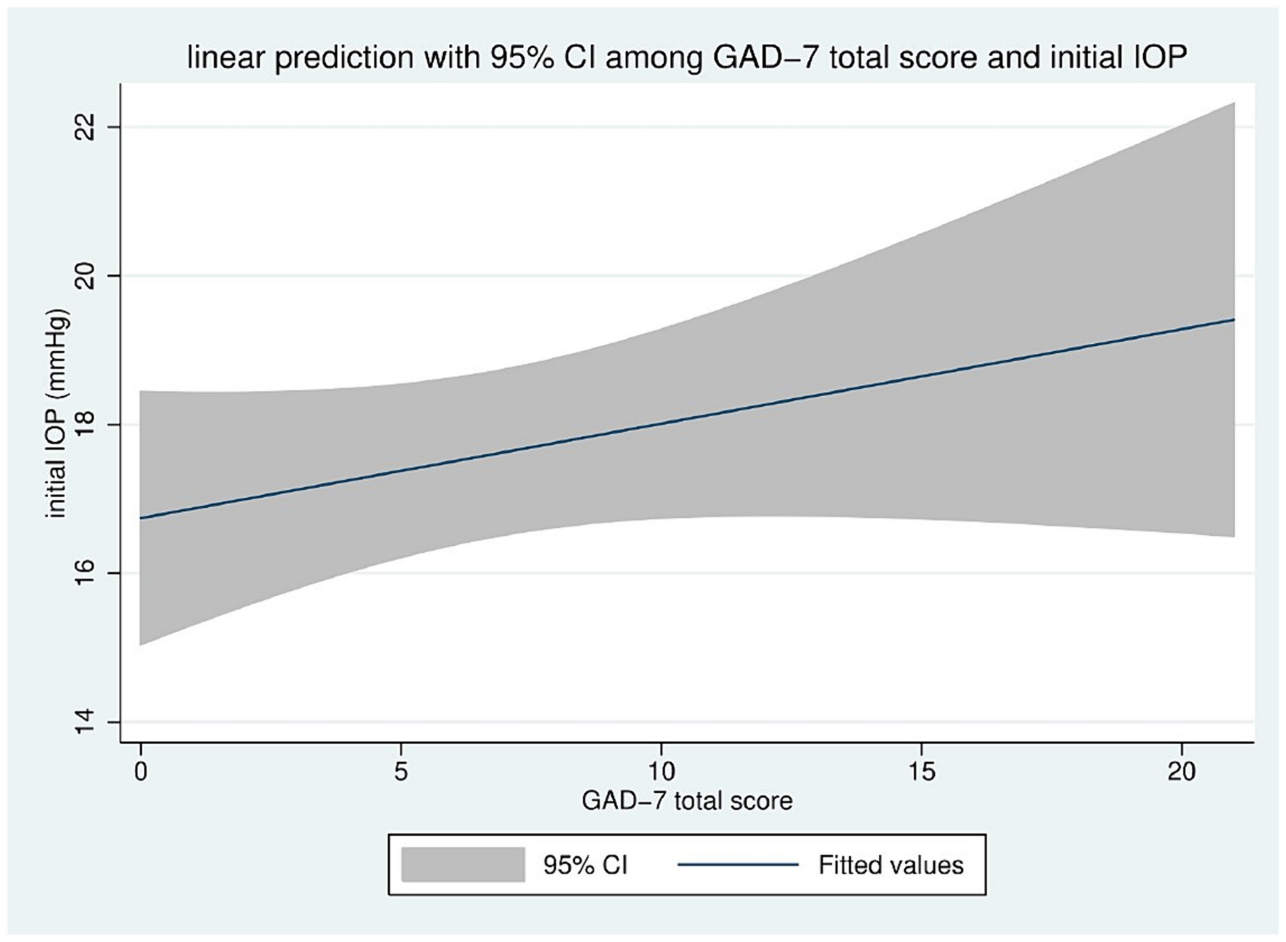
Linear correlation between GAD 7 total score and initial IOP.

The linear correlation between the total score of the GAD 7 and the initial IOP also shows a positive correlation, as seen in Figure 3. Among patients with GAD Anxiety scores ≥10, the mean age was 56.64 ± 19.76, the number of anti-glaucoma medications in use was 1.72 ± 1.26, and the mean intraocular pressure was 18.98 ± 8.34 mmHg. Table 5 reports all data analyzed in the study in the absence of depression (PHQ ≤ 10), presence of depression (PHQ >10), absence of anxiety (GAD ≤10), and presence of anxiety (GAD >10), concerning all critical variables.

**Table 5:** The report of all data analyzed in the study in the absence of depression (PHQ ≤ 10), presence of depression (PHQ > 10), absence of anxiety (GAD ≤10), and presence of anxiety (GAD >10).

	PHQ ≤ 10	PHQ > 10	*p*	GAD ≤ 10	GAD > 10	*p*
Age (y)	62.28 ± 14.41	59.81 ± 17.14	0.15	63.34 ± 12.89	56.64 ± 19.76	0.0024
Drugs (*n*)	1.65 ± 1.21	1.76 ± 1.36	0.29	1.66 ± 1.24	1.72 ± 1.26	0.39
IOP (mmHg)	18.47 ± 8.08	19.45 ± 8.15	0.22	18.64 ± 8.03	18.98 ± 8.34	0.40
**Hypertension**						
No, *n* (%)	90 (72.58)	34 (27.42)	0.70	89 (71.77)	35 (28.23)	0.37
Yes, *n* (%)	63 (75.00)	21 (27.42)		65 (77.38)	19 (22.62)	
**Diabetes**						
No, *n* (%)	110 (72.37)	42 (27.63)	0.48	111 (73.03)	41 (26.97)	0.54
Yes, *n* (%)	41 (77.36)	12 (22.64)		41 (77.36)	12 (22.64)	
**Cardiopathy**						
No, *n* (%)	130 (72.63)	49 (27.37)	0.38	127 (70.95)	52 (29.05)	0.006
Yes, *n* (%)	21 (80.77)	5 (19.23)		25 (96.15)	1 (3.85)	
**Education**						
Illiterate	2 (50.00)	2 (50.00)	0.038	3 (75.00)	1 (25.00)	0.12
First degree	40 (65.57)	21 (34.43)		41 (67.21)	20 (32.79)	
Second degree	32 (66.67)	16 (33.33)		32 (66.67)	16 (33.33)	
College	73 (83.91)	14 (16.09)		73 (83.91)	14 (16.09)	
Master	1 (100.00)	0 (0.00)		1 (100.00)	0 (0.00)	
Doctorae	1 (33.33)	2 (66.67)		2 (66.67)	1 (33.33)	
**Glaucoma strata, *n* (%)**						
Early	65 (73.03)	24 (26.97)	0.30	59 (66.29)	30 (33.71)	0.07
Moderate	34 (82.93)	7 (17.07)		34 (82.93)	7 (17.07)	
Severe	56 (70.00)	24 (30.00)		63 (78.75)	17 (21.25)	

## Discussion

Our data show that the prevalence of depression and anxiety is higher in patients with glaucoma compared to the Brazilian national average. Glaucoma is the leading cause of irreversible blindness worldwide and one of the most important causes of preventable blindness. Numerous factors are associated with the evolution to blindness in cases of glaucoma, including poor patient compliance, high drug costs, and adverse medication reactions. Comorbidities, such as Anxiety Disorder and Depressive Disorder, can discourage patients from treatment (Gusar et al., 2021; Shin et al., 2021; Stamatiou et al., 2022). Our study investigated the possible correlation of glaucoma with psychopathological symptoms such as anxiety or depression.

In this study sample, we included only patients diagnosed with glaucoma in different stages, excluding those without the disease. Most of the sample consisted of men over 60 diagnosed with Primary Open Angle Glaucoma, corroborating what was found in the general population with glaucoma.

In this study, the prevalence of depression was 35.48%, corroborating the literature that describes an incidence of depression in patients with glaucoma of 30% (Singaporean study) and 41.8% (South East Nigeria study). Our anxiety scores were higher than those of Gutenberg and his colleagues in the Brazilian population (34.61%). Therefore, it is reasonable to argue whether glaucoma can be a triggering factor for depression or anxiety.

The more impaired the visual field and visual function are, the more mental and behavioral disorders patients present, such as sleep disorders, stress, anxiety, and depression (Gusar et al., 2021; Shin et al., 2021; Stamatiou et al., 2022). Studies suggest a positive correlation between these psychopathologies and the severity of glaucoma, as in Mabuchi et al. (2008, 2012) and Ajith et al. (2022). Some other publications, Agorastos et al. (2013) and Abe et al. (2020), concluded that there is a correlation between glaucoma and psychopathologies.

Nigel Lim, in 2016, suggested that there are conditions that directly influence the prevalence of anxiety in the population with glaucoma, like increased optic nerve cup-to-disk ratio, reduced MD, and glaucoma staging (Lim et al., 2016).

This study showed a greater prevalence of depression (PHQ9 > 10) in patients with early and severe glaucoma and lower in patients with moderate glaucoma, respectively 26.97, 30, and 17.07%. This percentage is not directly related to a worse MD in the visual field.

The hypothesis for this finding is that people with glaucoma adapt to the environment and their condition, overcoming their difficulties and limitations. Which could mean that when newly diagnosed with early glaucoma, the patient suffers from the new perspective and begins a depressed mood. Over time, the patient with glaucoma would learn how to deal better with the problem and adapts to adversity.

In moderate glaucoma, it could be that the patient is more stabilized in his mood situation, adapted to his needs, and can manage his daily activities without suffering from the impact caused by both glaucoma and the presenting emotional quality of life.

With the worsening of the ophthalmological clinical condition and the possibility of imminent visual loss, the patient is more likely to be concerned about limiting his activities. Hence, their quality of life worsens, and limitations arise due to the inability to self-care, leading to a prospect of low quality of life, bringing anxious, depressive symptoms again, generating stress, which facilitates the establishment of depressive and anxious psychopathological conditions.

This study has some limitations. Only one group of patients diagnosed with glaucoma was included in their different degrees of severity without comparison with a control group with other ophthalmology conditions. Additionally, the questionnaires used to assess depressive (PHQ-9) and anxiety (GAD-7) symptoms are less complex than the comprehensive assessment instrument for Quality of Life (SF8) which is commonly used in other studies with the same investigative purpose.

Regarding Anxiety (GAD >10), this study found a significant correlation between patients with mild and severe glaucoma of 33.71 and 17.07%, respectively. Higher anxiety scores in patients with glaucoma may be attributed to emotional and environmental factors such as coping with the disease and changes in lifestyle and routine. With the worsening of glaucoma, impairment of daily activities, quality of life, and patient limitations arise. Regarding intermediate stages, the patient may adapt to all the changes in life; consequently, emotional symptoms such as anxiety, depression, anguish, stress, and fear would subside.

The high incidence of glaucomatous patients with undiagnosed and untreated psychiatric disorders impacts society. In this study, only 16 volunteers had a history of glaucoma-related psychopathologies. Fifty-five volunteers had moderate symptoms of depression after assessment by the PHQ-9, and fifty-four people were diagnosed with moderate anxiety symptoms by the GAD-7.

Our data, combined with others in the literature, reflect the need for a psychological approach to glaucoma patients; whose complaints could remain unnoticed by ophthalmologists. However, they expressed their inability to review their patient’s mental status due to a lack of skills and the unavailability of a specialized workforce in their institutes. Our data corroborate those found by AlSwailem et al. (2022), where ophthalmologists expressed their inability to review their patients’ mental status due to a lack of skills and the unavailability of specialized labor in their institutes.

## Conclusion

There is a correlation between glaucoma and a higher prevalence of anxiety and depression. In this study, symptoms and depressive symptoms of depression were correlated with glaucoma at a higher prevalence when compared to the general Brazilian population.

## Data Availability

The raw data supporting the conclusions of this article will be made available by the authors, without undue reservation.
