# Supplementary material for: Prevalence of anxiety and depression among patients with glaucoma
Source: Front Psychol. 2024 Aug 22;15:1410890. doi: 10.3389/fpsyg.2024.1410890 (PMC11378733; doi:10.3389/fpsyg.2024.1410890)
Supplement: Supplementary file 1 [file Data_Sheet_1.pdf]

APPENDIX:

Appendix 1: Patient Health Questionnaire (PHQ-9) in English (A) and Portuguese (B).

PATIENT HEALTH QUESTIONNAIRE-9 (PHQ-9)

**Over the <u>last 2 weeks</u>, how often have you been bothered by any of the following problems?** *(Use "✔" to indicate your answer)*	**Not at all**	**Several days**	**More than half the days**	**Nearly every day**
**1.** Little interest or pleasure in doing things	0	1	2	3
**2.** Feeling down, depressed, or hopeless	0	1	2	3
**3.** Trouble falling or staying asleep, or sleeping too much	0	1	2	3
**4.** Feeling tired or having little energy	0	1	2	3
**5.** Poor appetite or overeating	0	1	2	3
**6.** Feeling bad about yourself — or that you are a failure or have let yourself or your family down	0	1	2	3
**7.** Trouble concentrating on things, such as reading the newspaper or watching television	0	1	2	3
**8.** Moving or speaking so slowly that other people could have noticed? Or the opposite — being so fidgety or restless that you have been moving around a lot more than usual	0	1	2	3
**9.** Thoughts that you would be better off dead or of hurting yourself in some way	0	1	2	3

FOR OFFICE CODING *0* + ______ + ______ + ______

=Total Score: ______

If you checked off <u>any</u> problems, how <u>difficult</u> have these problems made it for you to do your work, take care of things at home, or get along with other people?

Not difficult at all	Somewhat difficult	Very difficult	Extremely difficult
☐	☐	☐	☐

Developed by Drs. Robert L. Spitzer, Janet B.W. Williams, Kurt Kroenke and colleagues, with an educational grant from Pfizer Inc. No permission required to reproduce, translate, display or distribute.

A

QUESTIONÁRIO SOBRE A SAÚDE DO/A PACIENTE- (PHQ-9)

**Durante as <u>últimas 2 semanas</u>, com que freqüência você foi incomodado/a por qualquer um dos problemas abaixo?** *(Marque sua resposta com "✔")*	**Nenhuma vez**	**Vários dias**	**Mais da metade dos dias**	**Quase todos os dias**
**1.** Pouco interesse ou pouco prazer em fazer as coisas	0	1	2	3
**2.** Se sentir "para baixo", deprimido/a ou sem perspectiva	0	1	2	3
**3.** Dificuldade para pegar no sono ou permanecer dormindo, ou dormir mais do que de costume	0	1	2	3
**4.** Se sentir cansado/a ou com pouca energia	0	1	2	3
**5.** Falta de apetite ou comendo demais	0	1	2	3
**6.** Se sentir mal consigo mesmo/a — ou achar que você é um fracasso ou que decepcionou sua família ou você mesmo/a	0	1	2	3
**7.** Dificuldade para se concentrar nas coisas, como ler o jornal ou ver televisão	0	1	2	3
**8.** Lentidão para se movimentar ou falar, a ponto das outras pessoas perceberem? Ou o oposto – estar tão agitado/a ou irrequieto/a que você fica andando de um lado para o outro muito mais do que de costume	0	1	2	3
**9.** Pensar em se ferir de alguma maneira ou que seria melhor estar morto/a	0	1	2	3

FOR OFFICE CODING *0* + ______ + ______ + ______

=Total Score: ______

Se você assinalou <u>qualquer</u> um dos problemas, indique o grau de <u>dificuldade</u> que os mesmos lhe causaram para realizar seu trabalho, tomar conta das coisas em casa ou para se relacionar com as pessoas?

Nenhuma dificuldade	Alguma dificuldade	Muita dificuldade	Extrema dificuldade
☐	☐	☐	☐

Desenvolvido pelos Drs. Robert L. Spitzer, Janet B.W. Williams, Kurt Kroenke e colegas, com um subsídio educacional da Pfizer Inc. Não é necessária permissão para reproduzir, traduzir, exibir ou distribuir.

B
