# Supplementary material for: Prevalence of anxiety and depression among patients with glaucoma
Source: Front Psychol. 2024 Aug 22;15:1410890. doi: 10.3389/fpsyg.2024.1410890 (PMC11378733; doi:10.3389/fpsyg.2024.1410890)
Supplement: Supplementary file 2 [file Data_Sheet_2.pdf]

Appendix 2: General Anxiety Disorder (GAD-7) in English (A) and Portuguese (B).

GAD-7

**Over the last 2 weeks, how often have you been bothered by the following problems?** *(Use ✓ " to indicate your answer)*	**Not at all**	**Several days**	**More than half the days**	**Nearly every day**
**1.** Feeling nervous, anxious or on edge	0	1	2	3
**2.** Not being able to stop or control worrying	0	1	2	3
**3.** Worrying too much about different things	0	1	2	3
**4.** Trouble relaxing	0	1	2	3
**5.** Being so restless that it is hard to sit still	0	1	2	3
**6.** Becoming easily annoyed or irritable	0	1	2	3
**7.** Feeling afraid as if something awful might happen	0	1	2	3

(*For office coding: Total Score T____ = ____ + ____ + ____ *)

B

Developed by Drs. Robert L. Spitzer, Janet B.W. Williams, Kurt Kroenke and colleagues, with an educational grant from Pfizer Inc. No permission required to reproduce, translate, display or distribute.

GAD-7

**Durante as últimas 2 semanas, com que freqüência você foi incomodado/a pelos problemas abaixo?** *(Marque sua resposta com "✔")*	**Nenhuma vez**	**Vários dias**	**Mais da metade dos dias**	**Quase todos os dias**
**1.** Sentir-se nervoso/a, ansioso/a ou muito tenso/a	0	1	2	3
**2.** Não ser capaz de impedir ou de controlar as preocupações	0	1	2	3
**3.** Preocupar-se muito com diversas coisas	0	1	2	3
**4.** Dificuldade para relaxar	0	1	2	3
**5.** Ficar tão agitado/a que se torna difícil permanecer sentado/a	0	1	2	3
**6.** Ficar facilmente aborrecido/a ou irritado/a	0	1	2	3
**7.** Sentir medo como se algo horrível fosse acontecer	0	1	2	3

(*For office coding: Total Score T____ = ____ + ____ + ____ *)

A

Desenvolvido pelos Drs. Robert L. Spitzer, Janet B.W. Williams, Kurt Kroenke e colegas, com um subsídio educacional da Pfizer Inc. Não é necessária permissão para reproduzir, traduzir, exibir ou distribuir.
