# Supplementary material for: Prevalence of anxiety and depression among patients with glaucoma
Source: Front Psychol. 2024 Aug 22;15:1410890. doi: 10.3389/fpsyg.2024.1410890 (PMC11378733; doi:10.3389/fpsyg.2024.1410890)
Supplement: Supplementary file 3 [file Data_Sheet_3.pdf]

Appendix 3: Scoring grid used with the chosen scales

GAD – 7

Valor total: 21
Interpretação.
Pontuações 5 (Ansiedade Leve)
Pontuações 10 (Ansiedade Moderada)
Pontuações 15 (Ansiedade Grave)
O GAD-7 nunca deve ser usado como único teste avaliador de ansiedade.
UMA **pontuação** de 10 ou mais no **GAD - 7** representa um ponto de corte razoável para identificar casos de **TAG** ou **GAD** (Generalization Anxiety Disease).
GAD - 7 é útil para triagem emocional, cuidados primários e saúde mental como uma ferramenta e medida dos sintomas para os quatro de transtornos de ansiedade mais comuns (Transtorno de Ansiedade Generalizada, Transtorno de Pânico, Fobia Social e Transtorno de Estresse Pós-Traumático).
Pontuações representam:
0-5 = leve
6-10 = moderado
11-15 = moderadamente grave.
16-21 = comprometimento e transtorno de ansiedade patológico.
Os direitos autorais pertencem à Pfizer Inc., mas o questionário é de domínio público.

PHQ-9
Pontuação total do PHQ-9: 27
Pontuações 5 (sintomas depressivos leves),
Pontuações 10 (sintomas depressivos moderados),
Pontuações 20 (sintomas depressivos graves)
A pontuação total varia de 0 a 27 e representa a soma das respostas dos nove itens.
A gravidade do quadro seria estimada conforme o seguinte:
0-4 pontos – sem depressão;
5-9 pontos – transtorno depressivo leve;
10-14 pontos – transtorno depressivo moderado;
15-19 pontos – transtorno depressivo moderadamente grave
20 - 27 pontos – transtorno depressivo grave.
PHQ-9 não é uma ferramenta diagnóstica e sim um instrumento norteador que auxilia o profissional de saúde na investigação do estado emocional do paciente avaliado.
